# Latent transitions across perceived parental marital conflict and family cohesion profiles in high school students

**DOI:** 10.3389/fpsyg.2022.954825

**Published:** 2022-11-16

**Authors:** Tingting Gao, Leilei Liang, Muzi Li, Yingying Su, Songli Mei, Chengchao Zhou, Xiangfei Meng

**Affiliations:** ^1^Center for Health Management and Policy Research, School of Public Health, Cheeloo College of Medicine, Shandong University, Jinan, Shandong, China; ^2^NHC Key Laboratory of Health Economics and Policy Research, Shandong University, Jinan, Shandong, China; ^3^School of Public Health, Jilin University, Changchun, Jilin, China; ^4^Department of Psychiatry, Faculty of Medicine and Health Sciences, McGill University, Montreal, QC, Canada; ^5^Douglas Research Centre, Montreal, QC, Canada

**Keywords:** perceived parental marital conflict, family cohesion, high school students, latent profile analysis, latent transition analysis

## Abstract

This study aimed to explore the latent profiles across perceived parental marital conflict and family cohesion, as well as the transition patterns within-person and within-sample profiles over time. We conducted a 1-year follow-up study with a sample of first-year high school students from China. A total of 453 participants were included in the present analysis. We identified the following three latent profiles: high parental conflict and poor family cohesion profile, moderate parental conflict and family cohesion profile, and low parental conflict and good family cohesion profile. Female students and those who not lived with parents together were more likely to perceive more parental marital conflict and less cohesion in the family. The majority of students with high transition probability remained in the same profiles over time. The counts of latent transition pattern also demonstrated that students remaining in the primary profile over time accounted for the large proportion. The present study advances empirical bases for confirming the family system theory’s notion that the family is not static, but dynamic. Findings provide the optimal timing of interventions toward healthy transition.

## Introduction

Family is one of the most influential and immediate ecological contexts for adolescents (Bronfenbrenner, 1992). The Chinese traditional culture emphasized family of the central importance to people, and Chinese people think highly of cooperation and harmony in the family (Ye et al., 2021). However, the conflict between parents is common and unavoidable in each family because of disagreements in various aspects (Cummings and Davies, 2002). Parental marital conflict is defined as a verbal or physical dispute and argument due to contradictions in family issues (Fincham, 1994). The prevalence of children exposed to interparental conflict is high and appears to be growing (Westrupp et al., 2015). The influence of parental marital conflict on individual’s growth and development during childhood has been highlighted, and this impact could continue to adolescence (Cummings et al., 2012). Moreover, adolescents are particularly subject to perceiving a conflict between their parents (Cummings and Davies, 2010). Most of the adolescents are not economically independent in China, which leave children to more rely on their parent for their livelihood (Xin et al., 2009). There are a considerable and growing body of research demonstrating that parental marital conflict has a range of detrimental effects on children, including internalizing (e.g., depression, loneliness, and anxiety) (Kumar and Mattanah, 2018; Ran et al., 2021) and externalizing (e.g., suicidality and delinquency) problems (Liu et al., 2016; Ai et al., 2017; van Dijk et al., 2020), and adjustment problems (e.g., lower academic achievement) (Ghazarian and Buehler, 2010).

Individuals with high family cohesion are more likely to experience familial warmth and support, more effective communication, and fewer conflicts with family members (Myerberg et al., 2019). Characterized by the emotional bonding among family members, family cohesion reflects the extent of commitment to help and support one another within the family (Tolan et al., 1997; Zerach et al., 2013). Family cohesion can be described as a global indicator of family support, which serves as a source of social support and functions as a buffer against problematic outcomes (Farrell and Barnes, 1993). It is widely demonstrated that family cohesion led to a reduction in internalizing (e.g., depression and suicide ideation) (Joel Wong et al., 2012; Guassi Moreira and Telzer, 2015) and externalizing (e.g., alcohol use) problems (Rabinowitz et al., 2016; Cano et al., 2018) as well as adjustment problems (e.g., lower academic achievement) (Jhang, 2017).

Family is not a stationary or unchanged system, but in dynamic changing (Combrinckgraham, 1985). Family system theory suggests that family members are interdependent, with the wellbeing of one member having direct and indirect effects on the functioning and wellbeing of other family members (Abrams, 1999). The spillover hypothesis posits that behavior, affect, and mood arising from marital conflict will transfer to other family subsystems, especially the parent–child subsystem (Erel and Burman, 1995). Most prior studies have widely discussed the relationship across myriad family variables using a variable-centered approach. Empirical research has documented a negative association between the change in interparental conflict in a family and changes in parent–adolescent relationships in the same family (Kouros et al., 2014; Mastrotheodoros et al., 2019), which might in turn influence the level of adolescents’ family cohesion. Martial relationship quality may co-occur with other relationship qualities in the same family (Fitzgerald et al., 2020). Thus, it is necessary to explore parental marital conflict and family cohesion simultaneously.

There is still not a commonly accepted standard on how to evaluate the diagnostic level of parental marital conflict and family cohesion. While latent profile analysis (LPA), a person-centered approach, could identify unique profiles and has advantages over accuracy classification. There is an increasing number of studies that identify some profiles of multiple family relationships and functioning. For instance, a four-profile model of interparental conflict was selected in divorcing families (Elam et al., 2016). Four latent profiles for family relationships measured by interparental conflicts and parent–adolescent attachment were identified, which consist of disengaged, cohesive, moderate, and conflictual families (Zhang et al., 2021). Cohesive, permissive, controlling/disengaged, and controlling/enmeshed subgroups for family functioning were identified (Al Ghriwati et al., 2017). The study on the co-occurrence of parental monitoring and parent–adolescent conflict among Latinx adolescents identified three typologies as follows: “high monitoring/low conflict”; “moderate monitoring/moderate conflict”; and “high monitoring/moderately high conflict.” This research also highlighted the importance of exploring family variables within-person and within-sample profiles. Previous empirical studies have mainly focused on the outcome of parental marital conflict (Ghazarian and Buehler, 2010; van Dijk et al., 2020) and family cohesion (Rabinowitz et al., 2016; Jhang, 2017). However, the related factors with latent profiles characterized by a set of indicators for perceived parental marital conflict and family cohesion have been largely ignored.

The interparental conflict has been confirmed to fluctuate and vary over time (Kouros et al., 2010). Parental marital conflict is not a constant, but a dynamic process, which makes variability in children exposure to this conflict over time (Cummings and Davies, 2010). Previous research revealed levels of family conflict among youth in Northern Ireland were a non-linear change over time (Cummings et al., 2016). Family system theory insists that family cohesion could change to cope with the development of adolescents over time (Bowen, 1986; Jaggers et al., 2015). Significant decreases in family cohesion have been identified during early to middle adolescence (Baer, 2002). However, family cohesion was found to exhibit increased changes during the college transition (Guassi Moreira and Telzer, 2015). The existing literature highlighted the importance of examining trajectories of change in family cohesion (Richmond and Stocker, 2006). Previous theoretical foundations and findings hold the view that family cohesion and parental marital conflict fluctuated and varied over time, while no studies have reported on the transition within latent profiles over time. How the variability of the movement will evolve with a particular focus on transition from one profile to another across parental marital conflict and family cohesion?

The following research questions would be addressed in this study: (1) to explore distinct profiles across perceived parental marital conflict and family cohesion; (2) to determine the correlates of unique profiles; (3) to examine the transitions patterns among latent profiles of perceived parental marital conflict and family cohesion. These findings could promote a better understanding of the time-varying nature of family and contribute to making preventive and intervention measures in order to improve the perceived parental marital conflict and family cohesion.

## Materials and methods

### Participants and procedures

Data used in the present study were drawn from a longitudinal study conducted between October 2017 and October 2018. Students enrolled in a high school located in Changchun, China were selected using a random cluster sampling method. Participants in the 10th grade at the beginning were followed up 1 year later. Respondents were asked to complete a questionnaire with three waves. Each wave was separated by 6 months. The first (October 2017), second (April 2018), and third (October 2018) time point were identified as Time 1 (T1), Time 2 (T2), and Time 3 (T3), respectively. The sample consisted of 453 participants after excluded cases with too many missing values in variables used in the present study. The detailed process of sample selection and this sample has been described in the previous studies for different research (Gao et al., 2020, 2021), while the main variables in the present study were not analyzed. Variables including baseline demographics of the sample, perceived parental marital conflict and parent–child cohesion at each time point were used in the present study. Respondents were largely girls (*n* = 290, 64.0%) and from one-child families (*n* = 330, 72.8%). The age of participants ranged from 12 to 16 years (mean = 15.07; *SD* = 0.46). The majority of students were living with parents together (*n* = 375, 83.1%).

The present study has received the ethical approval from the Research Ethics Committee of the first author’s affiliation. The informed consent was obtained from students and their parents at each data collection. We provided a unique code for every participant in order to researchers matches their responses in the whole follow-up study. Before data collection, students were reminded that participation was voluntary and their responses would be kept confidential. More importantly, the collected data were only used for research and it would not have any influence on participants´study and life.

### Measures

#### Children’s perceptions of interparental conflict

Perceived parental marital conflict was measured *via* the characters of conflict subscale from the Chinese version of children’s perceptions of interparental conflict (CPIC) (Chi and Xin, 2003), which was originally developed by Grych et al. (1992). The 17-item subscale comprises three dimensions including conflict frequency, conflict intensity, and conflict resolution. Response scored on a 4-point Likert scale ranging from 1 (never) to 4 (always). Items were summed for their level of perceived parental marital conflict with higher scores indicating more perceptions of interparental conflict. In the present study, this measure demonstrated good internal consistency at each time point (α_*t*1_ = 0.91, α_*t*2_ = 0.92, α_*t*3_ = 0.92).

#### Family cohesion

Family cohesion was measured by the cohesion subscale from the Chinese version of Family Adaptability and Cohesion Scale (FACES II-CV) (Fei et al., 1991), which was originally developed by Olson et al. (1982). The subscale of family cohesion includes 16 items, of which four are reverse-scored items. All items were assessed by a 5-point Likert scale with response options from 1 (never) to 5 (very often). Items were summed for the degree of participants’ emotional connection to their family with higher scores representing better family cohesion. This measure demonstrated internal consistency in the present study across three waves (α_*t*1_ = 0.89, α_*t*2_ = 0.89, α_*t*3_ = 0.92).

#### Covariates

Socio-demographic variables included age, sex (1 = female, 2 = male), whether from a one-child family (1 = yes, 2 = no), and living with parents together or not (1 = yes, 2 = no).

### Statistical analysis

We utilized the LPA to specify the latent profiles between perceived parental marital conflict and family cohesion at each study time point. As a person-centered technique, LPA assumes each individual belongs to one of the latent profiles and estimates the membership probability for each participant (Emm-Collison et al., 2020). Each individual was classified into a latent profile according to the membership probability. The optimal number of profiles was determined by a variety of model fit indices. A lower value of the Akaike information criterion (AIC), Bayesian information criterion (BIC), adjusted BIC (aBIC) suggests a better-fitting model (Tofighi and Enders, 2007). The classification accuracy is preferred with an entropy value closer to one (Clark, 2010). Considering the more meaningful classification, we did not consider the too small sample size of each latent profile with <5% of the sample. After comparison, a statistically significant Lo–Mendell–Rubin Likelihood Ratio Test (LMR-LRT) and Bootstrapped Likelihood Ratio Test (BLRT) indicate the (κ-1)-class model should be rejected to support a κ-class model (Nylund et al., 2007). Multinomial logistic regressions were adopted to determine the prediction of correlates on profiles of perceived parental conflict and family cohesion at baseline. Odds ratios for predictors of the latent profile were provided. Based on the identified number of latent profiles in each cross-sectional data, latent transition analysis (LTA) was applied to explore the stability and the transition patterns of perceived marital parental conflict and family cohesion subtypes between the two adjacent times across two transition points. Compared to transition patterns across several time points conducted in the repeated-measures latent class analysis (RMLCA), LTA has advantages over changes that occurs between the two adjacent times (Collins and Lanza, 2010). Measurement invariance in LTA was assumed to be equal across time considering the interpretability of latent classes over time (Collins and Lanza, 2010). Transition probability was reported to reflect that individuals would change among the latent profiles over time. The software has the assumption that the data are missing at random. Full-information maximum-likelihood (FIML) estimation was used to handle the missing. All the statistical analyses were conducted using the software of Mplus 8.3 (Muthén and Muthén, 1998-2017) and SPSS 25.0 (IBM Corporation, NY, USA).

## Results

### Latent profile models selection

A 3-profile solution was identified as the optimal LPA model for perceived parental marital conflict and family cohesion based on the lower values of AIC, BIC, aBIC, and significant *p*-value of LMR-LRT (see Table 1, for more details).

**TABLE 1:** Model fit statistics for the latent profile models of parental marital conflict and family cohesion at each time point.

Model	AIC	BIC	aBIC	Entropy	LMR-LRT (*P-value*)	BLRT (*P-value*)
**T1 (at baseline)**						
2-profile	4560.604	4614.111	4572.854	0.862	<0.001	<0.001
**3-profile**	**4350.649**	**4424.735**	**4367.610**	**0.863**	**<0.001**	**<0.001**
4-profile	4285.689	4380.354	4307.360	0.904	0.211	<0.001
5-profile	4218.504	4333.749	4244.887	0.810	0.691	<0.001
**T2 (6-month follow-up)**						
2-profile	4514.800	4568.306	4527.049	0.838	<0.001	<0.001
**3-profile**	**4292.894**	**4366.980**	**4309.855**	**0.858**	**0.006**	**<0.001**
4-profile	4229.893	4324.559	4251.565	0.808	0.081	<0.001
5-profile	4164.094	4279.339	4190.477	0.823	0.246	<0.001
**T3 (12-month follow-up)**						
2-profile	4481.478	4534.985	4493.727	0.853	<0.001	<0.001
**3-profile**	**4270.668**	**4344.754**	**4287.629**	**0.864**	**0.041**	**<0.001**
4-profile	4174.734	4269.400	4196.406	0.837	0.379	<0.001
5-profile	4073.283	4188.528	4099.666	0.851	0.073	<0.001

AIC, akaike information criterion; BIC, bayesian information criterion; aBIC, adjusted BIC; LMR-LRT, Lo–Mendell–Rubin likelihood ratio test; BLRT, bootstrapped likelihood ratio test.

The bold values represent the final model.

**FIGURE 1:**
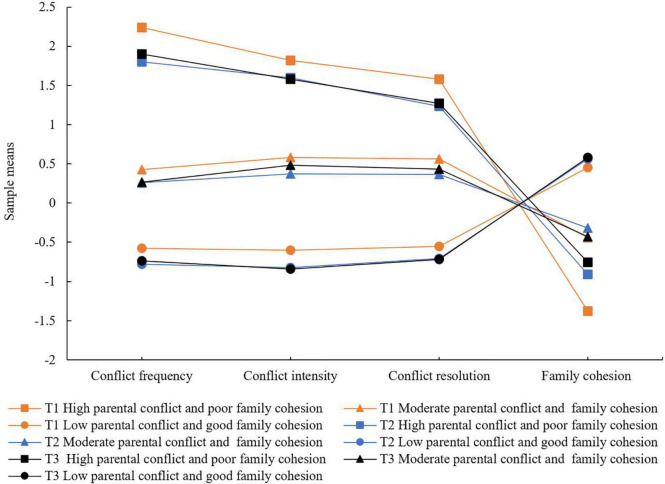
The sample means of latent profiles for parental marital conflict and family cohesion at each time point. The *y*-axis represents the mean score of parental marital conflict and family cohesion. All the dimensions are listed on the *x*-axis.

Figure 1 illustrates the sample means of three profiles for perceived parental marital conflict and family cohesion at each time point. The pattern was similar at all the three time points, indicating that the types of latent profiles remained unchanged over time. Students in “high parental conflict and poor family cohesion” profile engaged high mean scores (more than 1.8) on parental marital conflict indicators showing high conflict frequency, strong conflict intensity, and poor conflict resolution, while reported low means scores (less than –0.4) on family cohesion indicators showing poor family cohesion. Profiles are characterized by moderate mean scores (–0.5 to 0.6) of all dimensions labeled “moderate parental conflict and family cohesion.” Participants in the “low parental conflict and good family cohesion” profile had the smallest mean scores on all dimensions of parental marital conflict (less than –0.5) and highest mean scores on family cohesion (more than 0.4).

### The association between demographic characteristics and latent profiles at baseline

Multinomial logistic regression analysis was used to explore the predictors of perceived parental marital conflict and family cohesion latent profiles (Table 2). Using the “low parental conflict and good family cohesion” as the reference profile, results indicated female students were more likely to be the members of the high parental marital conflict and poor family cohesion profile as compared to their male counterparts (*p* < 0.05). Moreover, those not living with parents together were inclined to be classified into the moderate parental marital conflict and family cohesion profile (*p* < 0.01) and high parental marital conflict and poor family cohesion profile (*p* < 0.05).

**TABLE 2:** Multinomial logistic regression for the effects of correlates on the latent profiles.

	Moderate conflict and cohesion			High conflict and poor cohesion		
		
	B	SE	OR (95% CI)	B	SE	OR (95% CI)
**Gender**						
Male	Ref.			Ref.		
Female	–0.24	0.21	0.79 (0.52–1.20)	0.86	0.42	2.37 (1.04–5.40)*
**Living with parents together**						
Yes	Ref.			Ref.		
No	0.72	0.27	2.05 (1.20–3.49)**	0.97	0.42	2.63 (1.17–5.95)*

*p < 0.05; **p < 0.01.

### Changes in profiles for perceived parental marital conflict and family cohesion over time

The probabilities and number of students transitioning across profiles for parental marital conflict and family cohesion are presented in Table 3. There was the least proportion of students in the high parental conflict and poor family cohesion profile across the study period. The proportion of high parental conflict and poor family cohesion profiles increased over time. Although a little increase in proportion from T2 to T3, the overall proportion of low parental conflict and good family cohesion profile accounted for the largest proportion and declined across time. The majority of students stayed in the same profiles over time with the transition probability of parental marital conflict and family cohesion ranging from 0.681 to 0.902. A higher transition probability was found in the change from high parental conflict and poor family cohesion profile to moderate parental conflict and family cohesion profile. Besides, the transition from low parental conflict and good family cohesion profile to moderate parental conflict and family cohesion profile also claimed our attention. A higher transition probability indicated that variability occurs in two profiles with the mean scores next to each other.

**TABLE 3:** Latent profile proportion and transition probabilities for the latent transition analysis (LTA) model.

	Latent profiles		
	
Latent profile proportion	Low conflict and good cohesion	Moderate conflict and cohesion	High conflict and poor cohesion
T1	217 (47.9%)	186 (41.1%)	50 (11.0%)
T2	206 (45.5%)	192 (42.4%)	55 (12.1%)
T3	208 (45.9%)	189 (41.7%)	56 (12.4%)
Transition probabilities^#^	T2 (T3)		
**T1 (T2)**			
Low conflict and good cohesion	**0.867 (0.902)**	0.115 (0.086)	0.018 (0.013)
Moderate conflict and cohesion	0.071 (0.097)	**0.841 (0.822)**	0.089 (0.082)
High conflict and poor cohesion	0.052 (0.074)	0.240 (0.245)	**0.708 (0.681)**

^#^Transition matrix from T1 (T2) to T2 (T3); rows for T1 (T2), columns for T2 (T3). Transition probabilities in bold indicate the stability of profiles from one-time point to the next.

**FIGURE 2:**
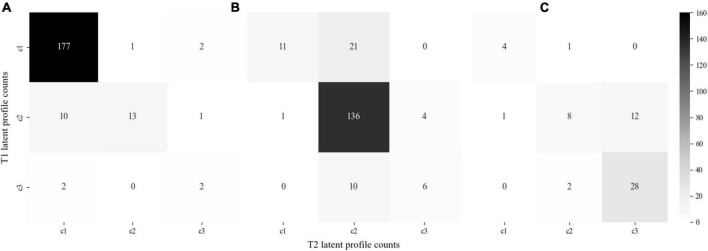
The profile counts of latent profile pattern for perceived parental marital conflict and family cohesion across three time points. c1: low parental marital conflict and good family cohesion profile; c2: moderate parental marital conflict and family cohesion profile; and c3: high parental marital conflict and poor family cohesion profile. The *y*-axis represents T1 latent profile counts, while the *x*-axis indicates counts of students moving to the T2 latent profile. **(A)** Counts of participants moving to the c1 profile at T3; **(B)** counts of participants moving to the c2 profile at T3; and **(C)** counts of participants moving to the c3 profile at T3.

The profile counts of latent profile patterns for perceived parental marital conflict and family cohesion over time are displayed in Figure 2. The majority of respondents stayed in the same profile over time. The low parental marital conflict and good family cohesion profile accounted for the biggest proportion (*n* = 177, 39.1%), followed by moderate parental conflict and family cohesion profile (*n* = 136, 30.0%) and high parental conflict and poor family cohesion profile (*n* = 28, 6.2%) across the studied three time points. It is worth noting that approximately 4.6% (*n* = 21) of students were classified into low parental marital conflict and good family cohesion profile at T1 and moved to moderate parental conflict and family cohesion profile at T2 and T3.

## Discussion

The present study explored perceived parental marital conflict and family cohesion using person-centered methods in Chinese high school students over a 1-year period. The results better explained the nature and significant heterogeneity in perceived parental marital conflict and family cohesion in Chinese culture. We identified the following three latent profiles: high parental conflict and poor family cohesion profile, moderate parental conflict and family cohesion profile, and low parental conflict and good family cohesion profile. Based on this classification, the socio-demographic correlates of profile membership between perceived parental marital conflict and family cohesion were determined. Our findings also supported perceived parental marital conflict and family cohesion would change over time.

The present study identified three discrete latent profiles across parental marital conflict and family cohesion with similar patterns at all the three time points, which was different from the previous research on parental conflict with other family variables (Elam et al., 2016; Zhang et al., 2021). Three profiles of non-residential father engagement (i.e., support to the adolescent, contact frequency, remarriage, relocation, and interparental conflict) were identified in divorced fathers (Modecki et al., 2015). In addition, prior work found four profiles for family functioning based on family cohesion, expressiveness, conflict, organization, and control (Al Ghriwati et al., 2017). Students who were classified into the high parental conflict and poor family cohesion profiles perceived more parental marital conflict but poor family cohesion. In contrast, low parental conflict and good family cohesion profiles represented low levels of parental marital conflict and high levels of family cohesion. Students in moderate parental conflict and family cohesion profile exhibited moderate levels of perceived parental marital conflict and family cohesion.

We found female students were at heightened risk for high parental marital conflict and poor family cohesion than males. Physical differentiation and conventional gender-differentiated social roles make women and men differ in many aspects. Boys are encouraged to exhibit more independence, self-direction, self-protection, and autonomy. In contrast, girls are good at communication within social networks. However, more communal dispositions may increase susceptibility to being more reactive to the risk of interparental conflict than their male peers (Davies and Lindsay, 2004). In addition, girls depend on emotional support from social relationships and are more sensitive to interpersonal stressors than boys (Rudolph, 2002; Guassi Moreira and Telzer, 2015). The parental marital conflict that results in adverse changes in family cohesion seems to be more salient for girls. Not living with parents together increased participant’s vulnerability to perceive more parental marital conflict and less family cohesion. The reason why not living with parents together might be parental separation, divorce, remarriage, death, and other causes regarding alienation of mutual affection. Children might be vulnerable to witnessing or exposure to parental conflict when they lived in a household with their parents. Spatial distance is also a potential factor disrupting family solidarity, due to limiting parents providing help and support for children, which results in poor family cohesion for students.

Most of the students were classified into low parental marital conflict and good family cohesion profiles. As expected, respondents perceiving high parental marital conflict and poor family cohesion accounted for the lowest proportion. The proportion of participants perceiving high parental conflict and poor family cohesion profile increased across the study period, while the overall decline in the proportion of low parental conflict and good family cohesion profile was found. Previous research reported a decreasing trend in levels of family cohesion as adolescents develop (Baer, 2002). The transition probability revealed that over two-thirds of students remained in the same profile over time. At least 68.1% of individuals could not change their high perceived parental conflict and poor cohesion to the family without receiving any specific intervention. The transition from high parental conflict and poor family cohesion profile to moderate parental conflict and family cohesion profile was found a higher probability. It is noteworthy that a relatively proportion of individuals could transition from low parental conflict and good family cohesion profile to moderate conflict and cohesion profile. Interventions were made to promote a healthy transition and prevent bad alterations.

The counts of latent transition pattern also demonstrated that students remaining in the primary profile over time accounted for the large proportion of low parental marital conflict and good family cohesion profiles. Moreover, the whole change across profiles is not obvious. Prior research found adolescents exhibit stability in family cohesion because they are still living with family during early and middle adolescence (Constante et al., 2019). The prompt interventions targeted on students with a high perception of parental marital conflict and poor family cohesion should be made in order to prevent the persistence of problems. Approximately 4.6% (*n* = 21) of students were classified into low parental marital conflict and good family cohesion profile at baseline, then they moved to moderate parental conflict and family cohesion profile at T2 and T3. The transition from T1 to T2 is an important intervention time point. If students start to perceive moderate parental conflict and family cohesion after this transition time point, they might keep this status for a long time. Because they will not change this status in the next research time point. Parents should solve marital problems by communication rather than conflict, which will decrease children’s perception of parental discord. In addition, spending more time on involvement in family activities and children’s lives is also necessary for parents. Pay more attention to children’s emotional needs and increase their belongingness to family.

### Limitations and future directions

There are several limitations in the present study. The first-year students recruited from one high school were included in our research. It is necessary to replicate our results on a more representative sample in the future. Moreover, there are lots of covariates correlated to perceived parental conflict and family cohesion. Besides the studied variables, more related factors should be included in the future study. In addition, the existing literature mainly focused on parental conflict and family cohesion using the person-centered method is limited, which results in our findings lack of comparison with others. Further research on the classification, the effect of correlates on profile membership, and changes over time in terms of perceived parental marital conflict and family cohesion using person-centered approaches need to be explored in the future.

### Practical implications

This study advanced a person-oriented analysis to identify the profile membership, which revealed the heterogeneity and nature of perceived parental marital conflict and family cohesion. According to the response to each indicator, we could understand the characteristics of individuals with high perceived parental conflict and poor family cohesion. More targeted interventions can be made for the highest-risk group. Screening of at-risk individuals exposed to more correlates of high parental conflict and poor family cohesion contributes to adopt preventive measures for this population. The high possibility of being the same profile and large proportion over time stresses the need for interventions targeted at participants perceiving high parental marital conflict and poor family cohesion. Make full use of the transition from high parental conflict and poor family cohesion profile, low parental conflict and good family cohesion profile to moderate parental conflict and family cohesion profile. Strategies regarding the optimal timing of interventions toward healthy transition could be included.

## Conclusion

The present study provides a window into distinct patterns of characteristics and offers insight into the transitions among distinct profiles between perceived parental marital conflict and family cohesion in Chinese high school students. We identified three unique profiles between parental marital conflict and family cohesion based on a series of indicators by LPA. Female students and those who not living with parents together were more likely to perceive more parental marital conflict and less cohesion in the family. The transition patterns across profile membership between parental marital conflict and family cohesion within-person and within-sample profile over time was explored by LTA. The present study advances empirical bases for confirming family system theory’s notion that the family is not static, but dynamic (Combrinckgraham, 1985). In addition, being in the same profile for the parental conflict and family cohesion was relatively stable and accounted for the biggest proportion over time. Findings also provide the optimal timing of interventions toward healthy transition.

## Data availability statement

The raw data supporting the conclusions of this article will be made available by the authors, without undue reservation.

## Ethics statement

The studies involving human participants were reviewed and approved by Research Ethics Committee of Jilin University. Written informed consent to participate in this study was provided by the participants or their legal guardian/next of kin. Written informed consent was obtained from the individual(s) and minor(s)’ legal guardian/next of kin, for the publication of any potentially identifiable images or data included in this article.

## Author contributions

SM and TG designed the study. LL and TG performed the study. TG, ML, and YS analyzed the data and drafted the manuscript. XM, SM, and CZ participated in revising the manuscript. All authors approved the final manuscript.
